# Supplementary material for: Paths to adaptation under fluctuating nitrogen starvation: The spectrum of adaptive mutations in Saccharomyces cerevisiae is shaped by retrotransposons and microhomology-mediated recombination
Source: PLoS Genet. 2023 May 16;19(5):e1010747. doi: 10.1371/journal.pgen.1010747 (PMC10218751; doi:10.1371/journal.pgen.1010747)
Supplement: S1 Fig — Barcode trajectories during pooled fitness remeasurement experiments, A) in nitrogen limiting conditions, and B) in glucose limiting conditions. A subset of known neutral lineages is represented by dotted lines. Lineages in red have an estimated fitness >0.01, in grey between -0.01 and +0.01, and in blue < -0.01. (PDF) [file pgen.1010747.s001.pdf]

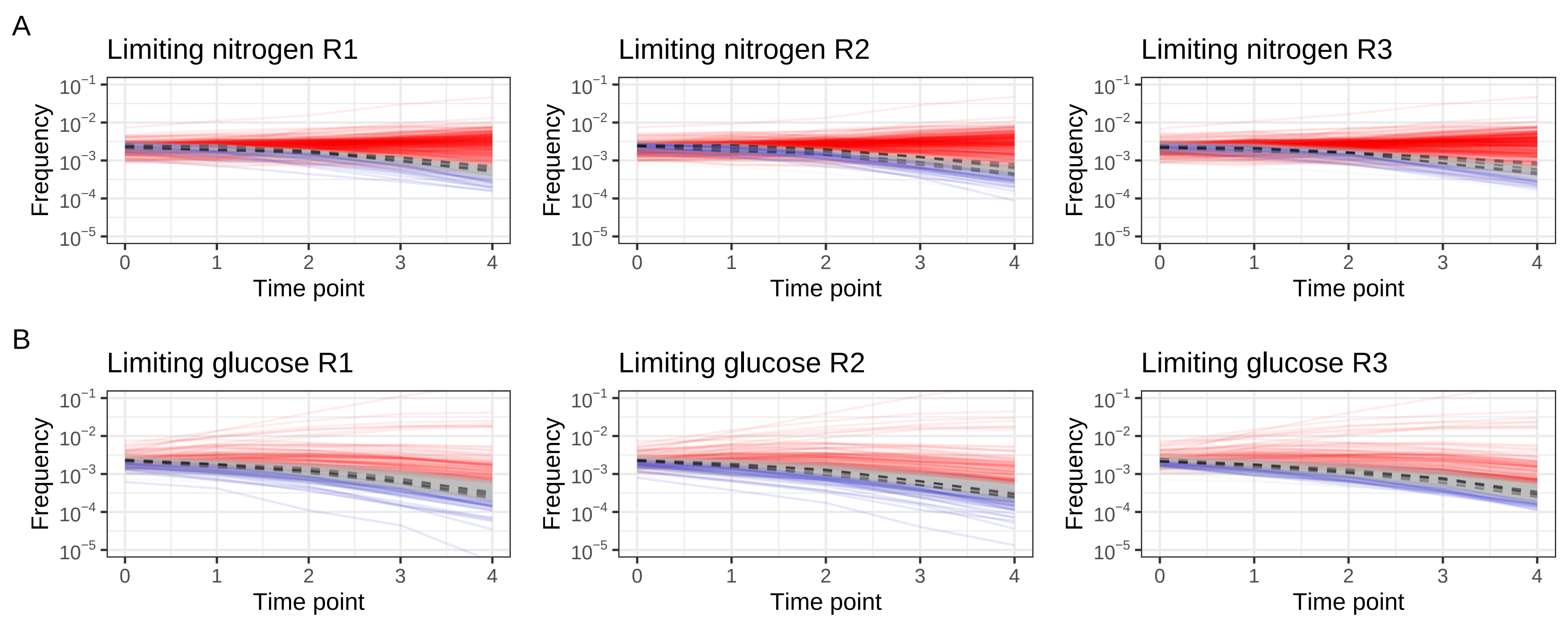
A
Limiting nitrogen R1
Limiting nitrogen R2
Limiting nitrogen R3
B
Limiting glucose R1
Limiting glucose R2
Limiting glucose R3
Frequency
Time point
10^{-1}
10^{-2}
10^{-3}
10^{-4}
10^{-5}
0
1
2
3
4
