# Supplementary figures and images for: Paths to adaptation under fluctuating nitrogen starvation: The spectrum of adaptive mutations in Saccharomyces cerevisiae is shaped by retrotransposons and microhomology-mediated recombination
Source: PLoS Genet. 2023 May 16;19(5):e1010747. doi: 10.1371/journal.pgen.1010747 (PMC10218751; doi:10.1371/journal.pgen.1010747)

A

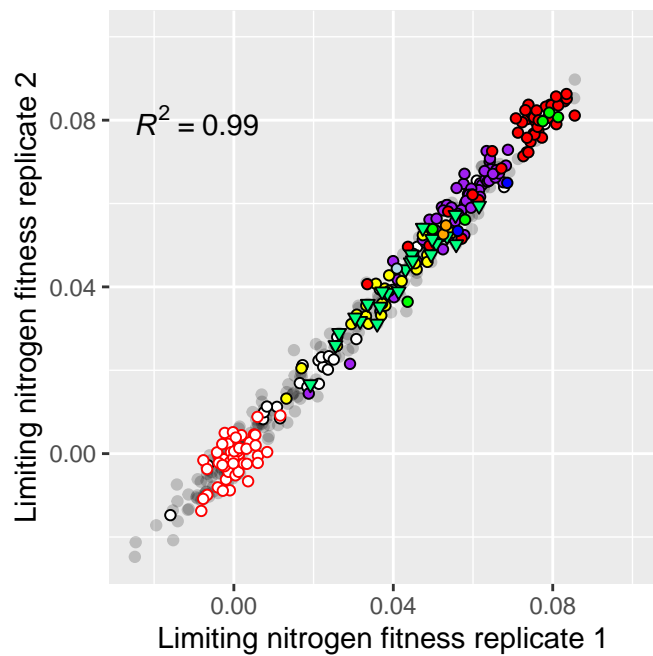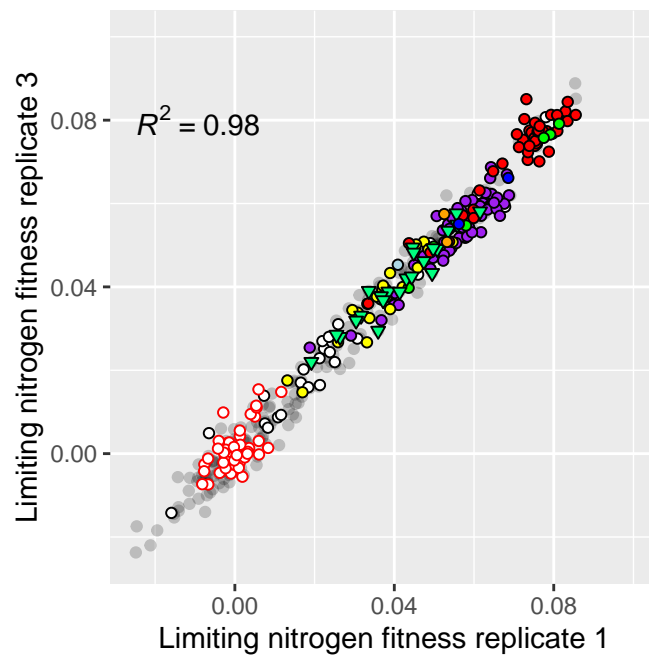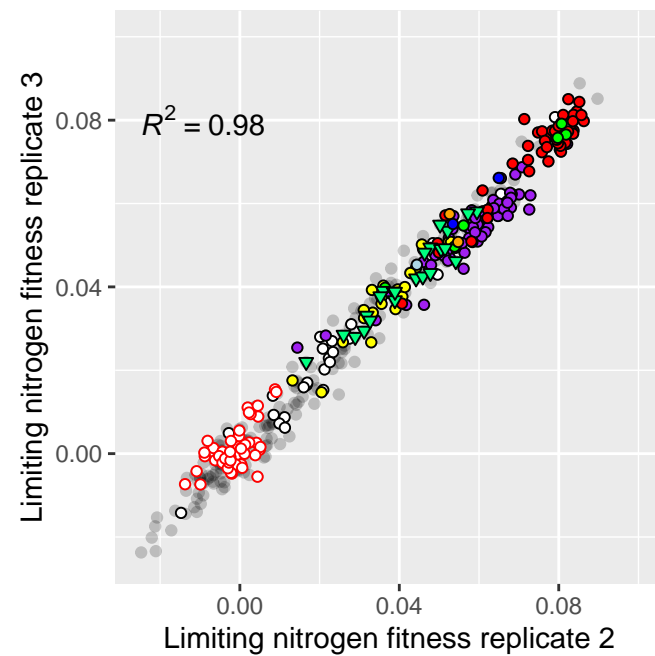

B

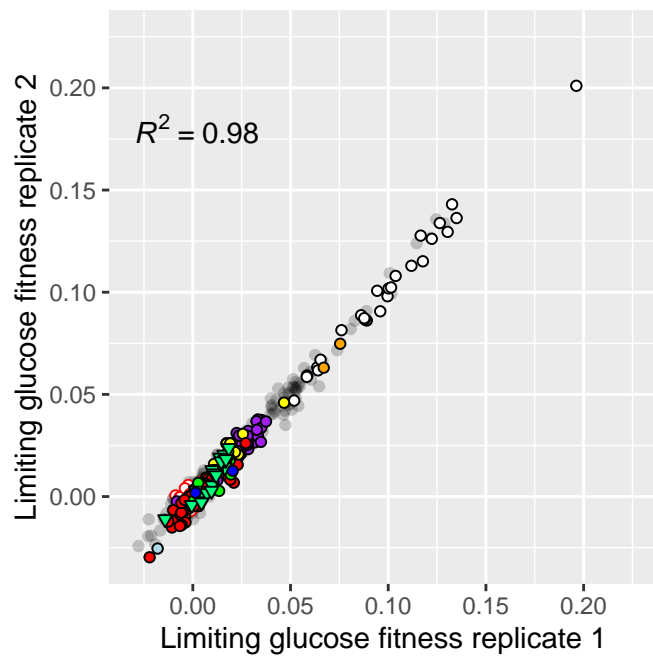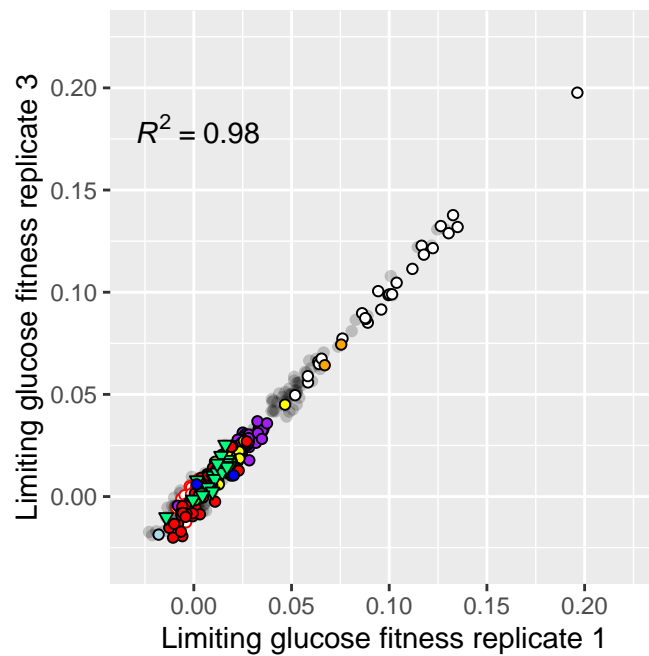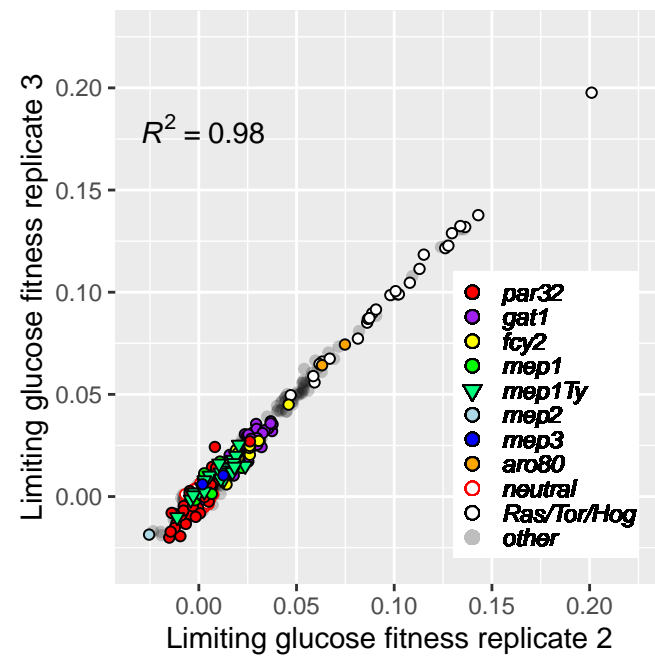

Supplement: S2 Fig — Estimated fitness between replicates for each clone under A) nitrogen limitation, and B) glucose limitation. (PDF) [file pgen.1010747.s002.pdf]

A.

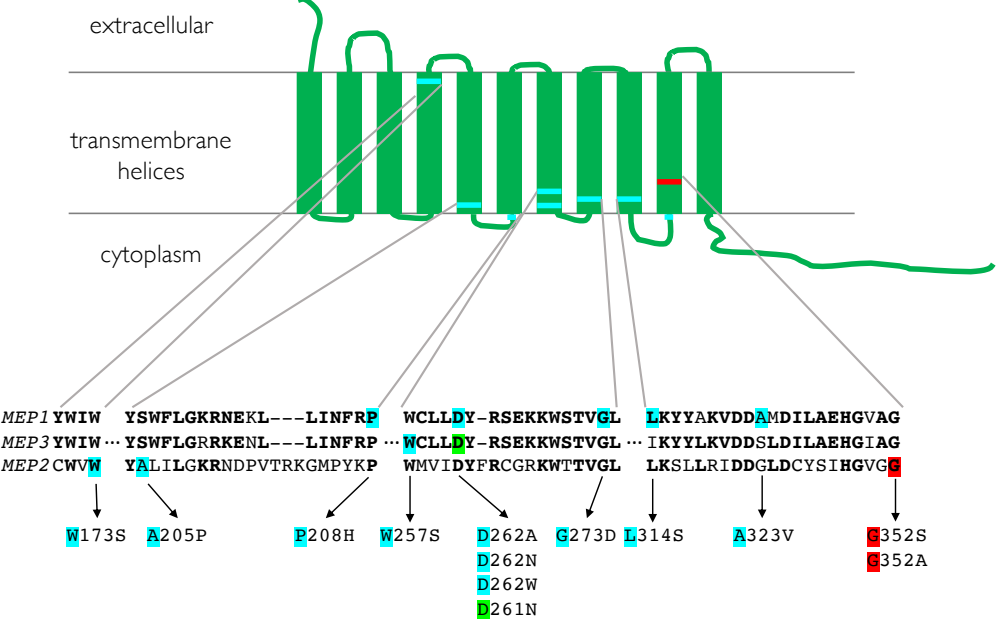

B.

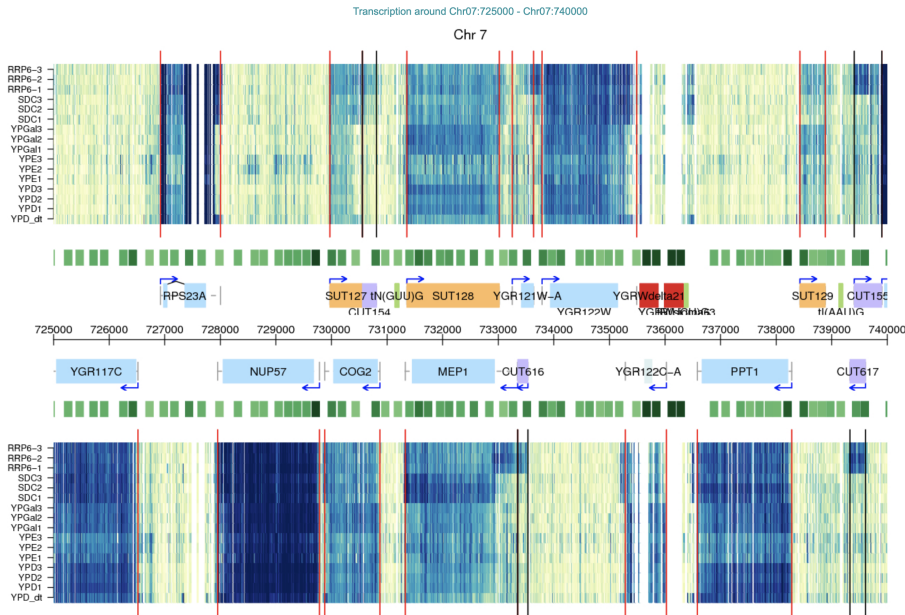

Supplement: S3 Fig — A) Locations of Mep mutations in a multiple alignment; the green highlighted residue identifies which gene had that mutation, while the red highlighted residues were mutations observed previously [4], and B) transcription at MEP1 locus indicates the presence of an antisense transcript downstream of MEP1. (PDF) [file pgen.1010747.s003.pdf]

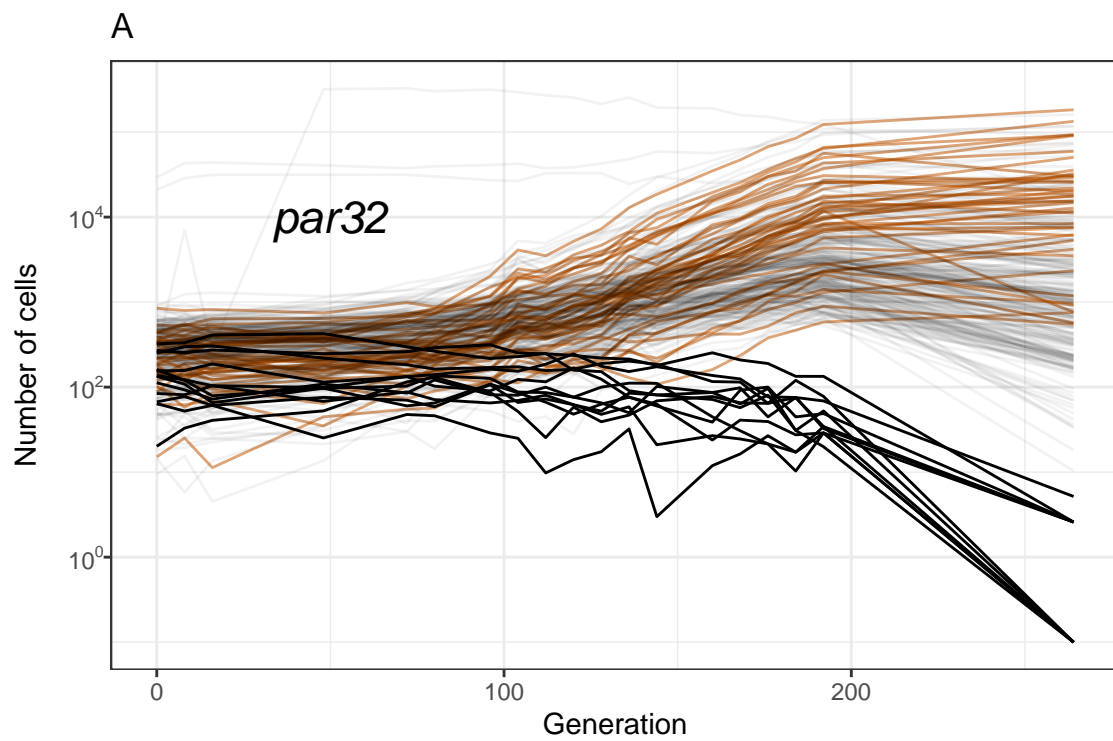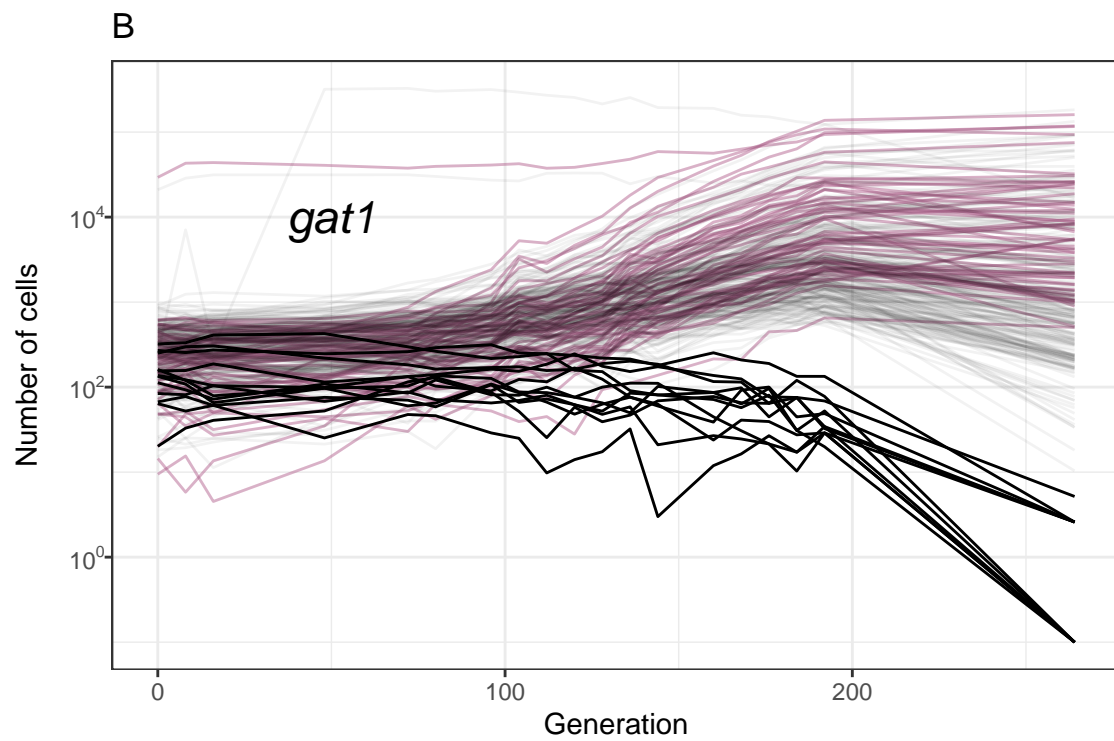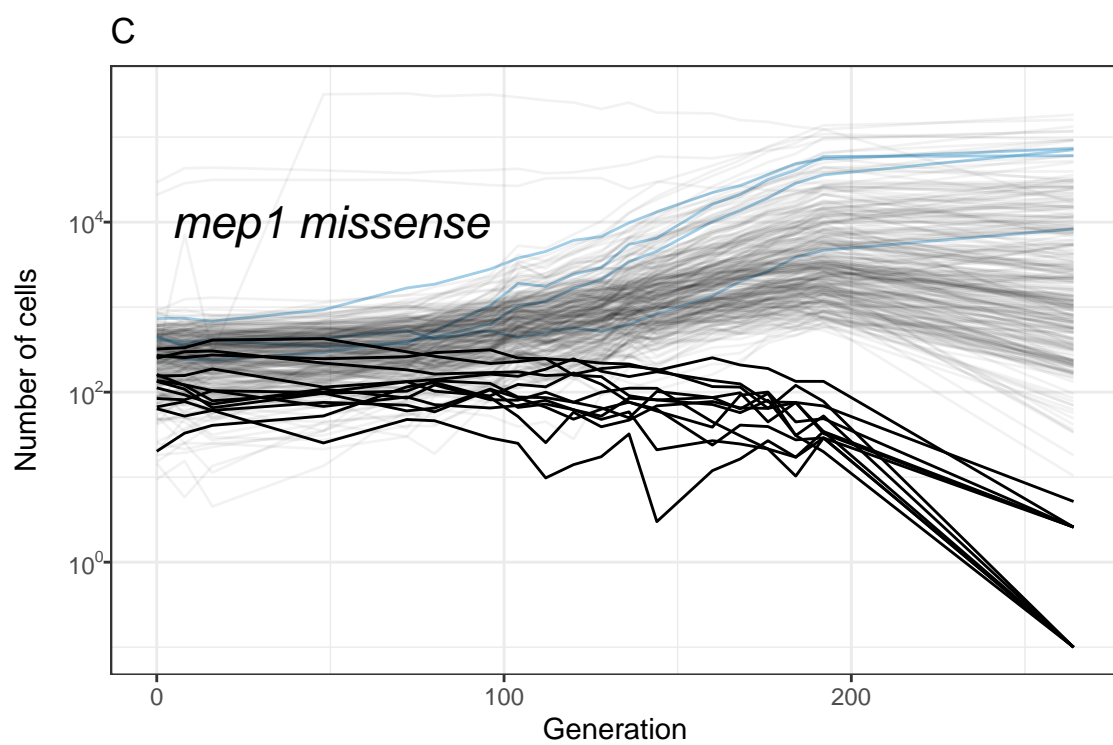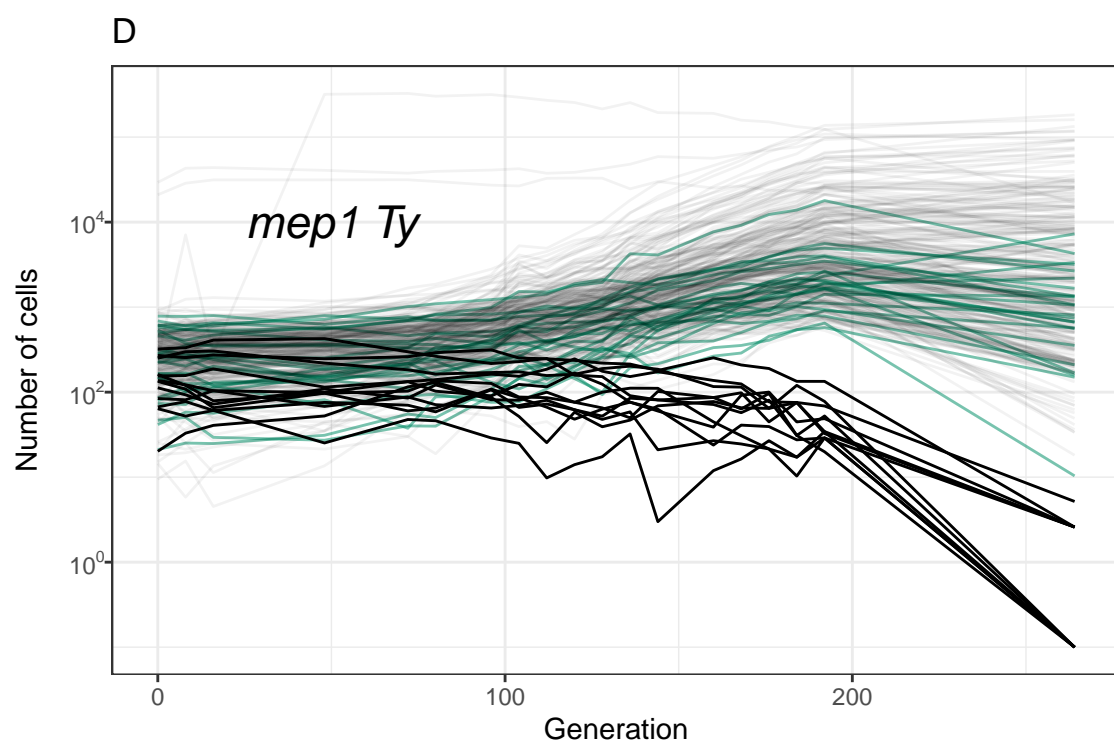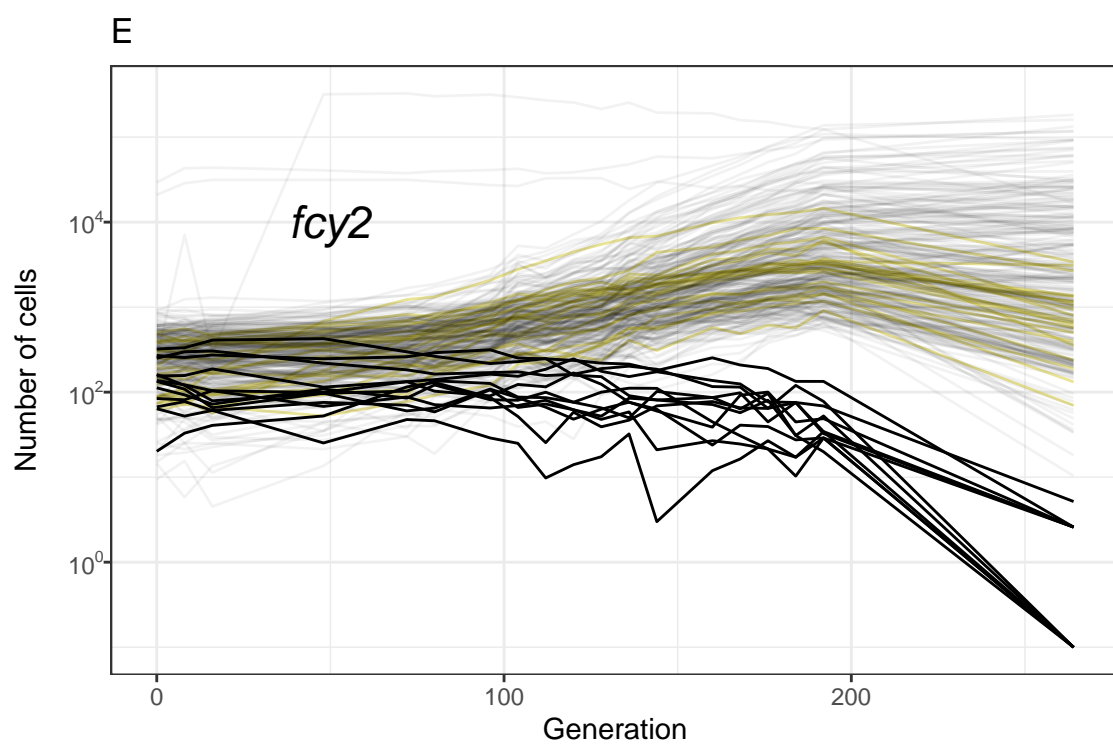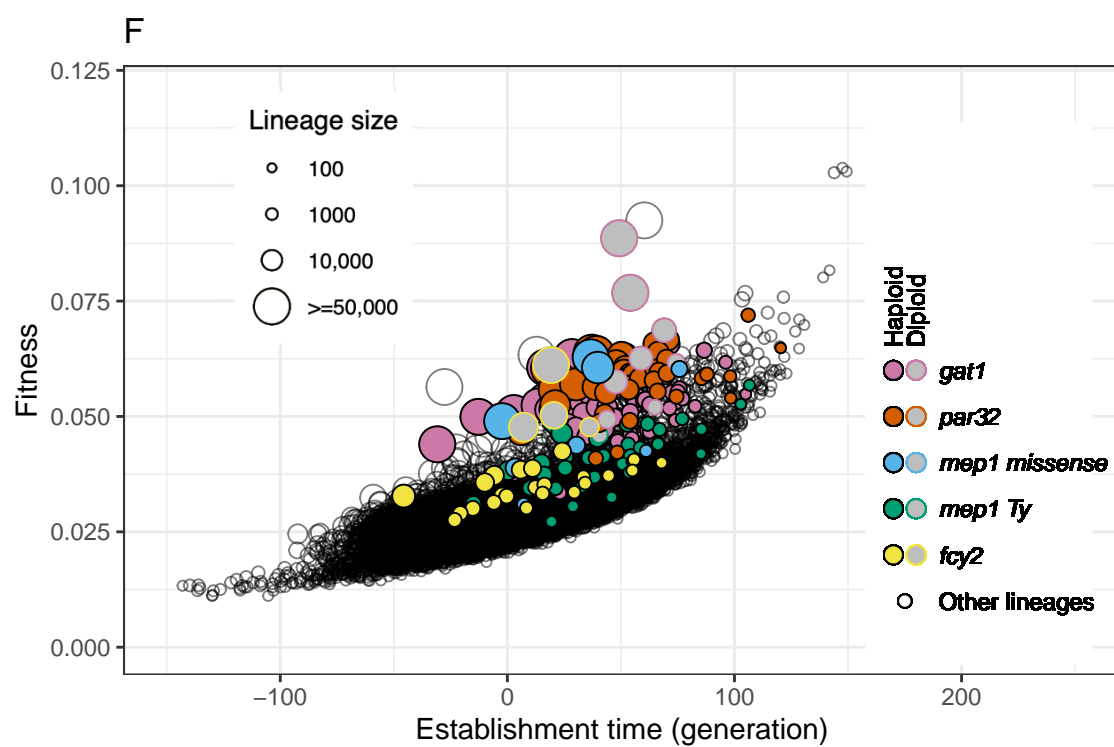

Supplement: S4 Fig — A) PAR32 (red), B) GAT1 (purple), C) MEP1 missense mutations (light blue and burgundy), D) MEP1 Ty1/2 insertions (green), E) FCY2 (yellow), and F) fitness and lineage size as a function of establishment time (color-coded the same way as panels A-E). Black lines in A-E denote neutral lineages. (PDF) [file pgen.1010747.s004.pdf]

**A**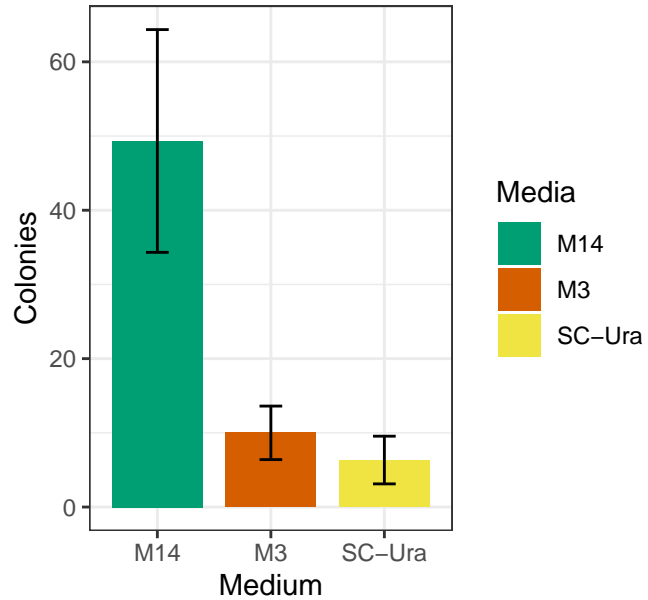**B**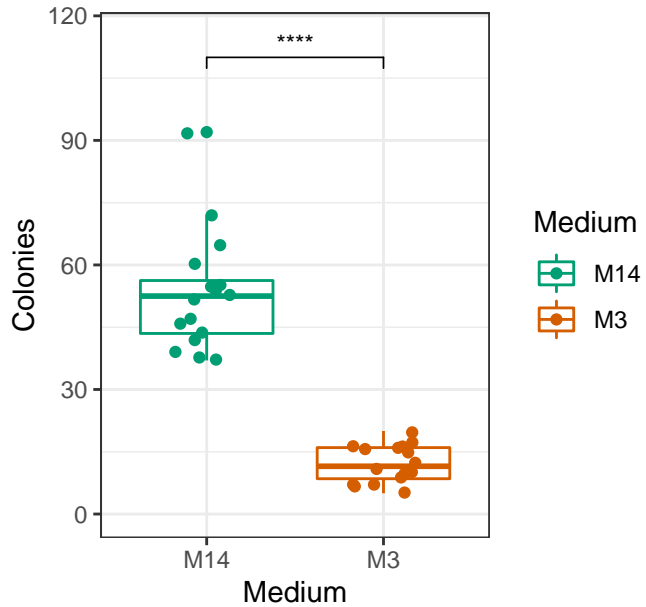

Supplement: S5 Fig — (A) Bars represent average of three WT strains with a transposon reporter plasmid; each value is the number of colonies on SC-His medium with each His+ colony representing independent Ty transposition events. Strains were grown in SC-Ura and then shifted to M14, M3 or SC-Ura as a control for 48 hours and plated on SC-His plates. (B) One WT strain with pGS234 was subjected to a fluctuation test (16x 5ml tubes) in M14 and M3 media. Each dot represents the number of colonies on each SC-His plate. Kruskal-Wallis chi-squared = 23.341, df = 1, p-value = 1.357e-06. (PDF) [file pgen.1010747.s005.pdf]

A

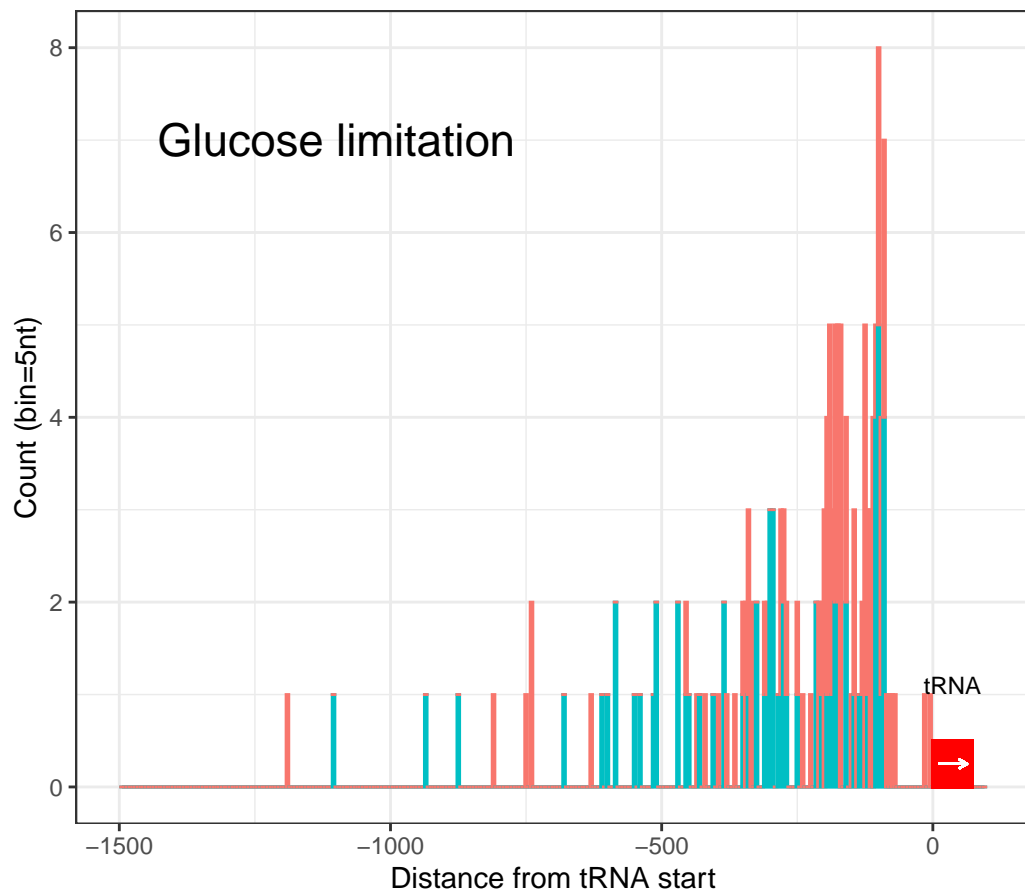

B

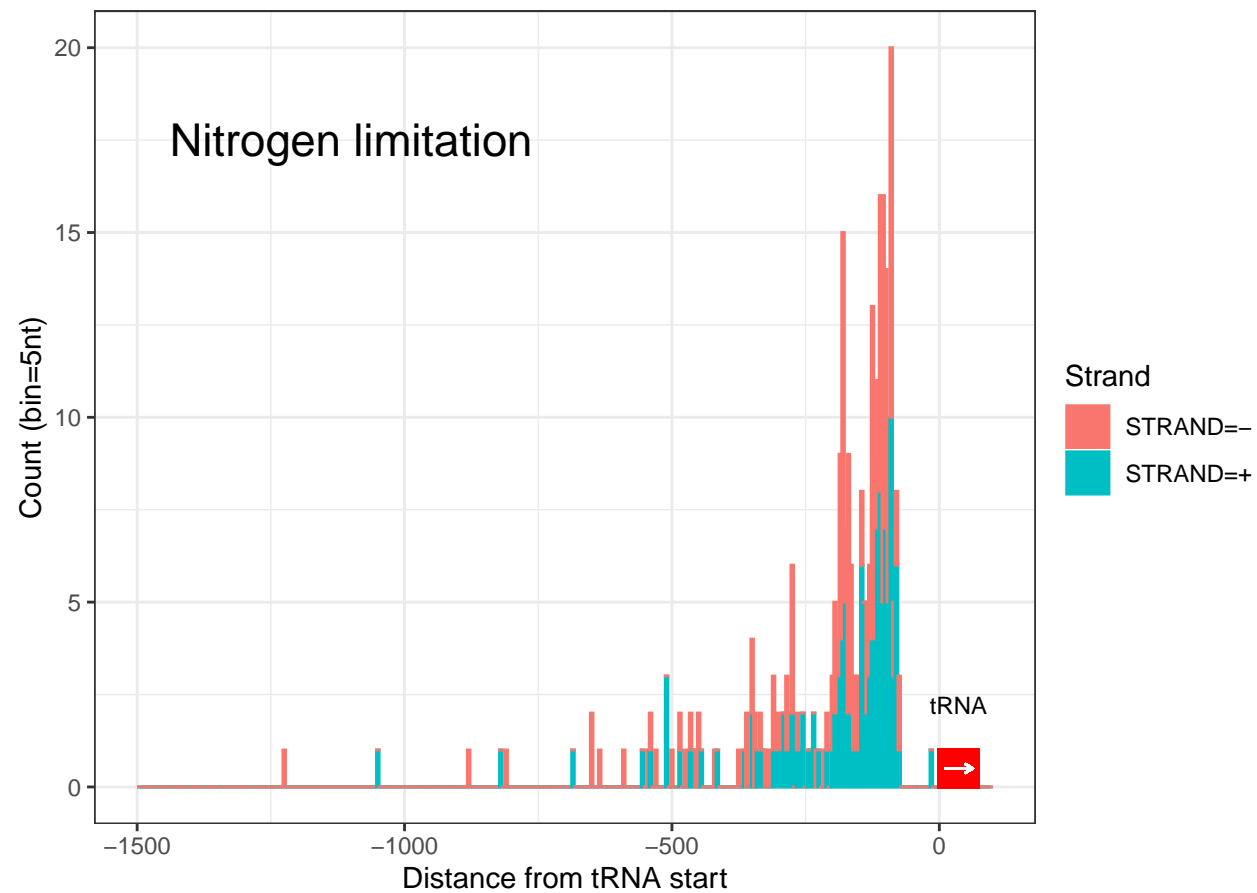

Supplement: S6 Fig — Insertion locations of Ty1/2 elements upstream of tRNA genes in A) glucose limitation and B) nitrogen limitation. New Ty1/2 element insertions typically prefer to land upstream of RNA Pol III transcribed elements, such as tRNAs [75, 79, 112]. (PDF) [file pgen.1010747.s006.pdf]
