# Supplementary material for: Paths to adaptation under fluctuating nitrogen starvation: The spectrum of adaptive mutations in Saccharomyces cerevisiae is shaped by retrotransposons and microhomology-mediated recombination
Source: PLoS Genet. 2023 May 16;19(5):e1010747. doi: 10.1371/journal.pgen.1010747 (PMC10218751; doi:10.1371/journal.pgen.1010747)
Supplement: S1 Table — (DOCX) [file pgen.1010747.s007.docx]

| F1 | ACACTCTTTCCCTACACGACGCTCTTCCGATCTAACACCTATTAATATGGACTAAAGGAGGCTTTT |
| --- | --- |
| F2 | ACACTCTTTCCCTACACGACGCTCTTCCGATCTACTAACTGTTAATATGGACTAAAGGAGGCTTTT |
| F3 | ACACTCTTTCCCTACACGACGCTCTTCCGATCTATCGCCAGTTAATATGGACTAAAGGAGGCTTTT |
| F4 | ACACTCTTTCCCTACACGACGCTCTTCCGATCTCATTCCAATTAATATGGACTAAAGGAGGCTTTT |
| F5 | ACACTCTTTCCCTACACGACGCTCTTCCGATCTCGCATTAATTAATATGGACTAAAGGAGGCTTTT |
| F6 | ACACTCTTTCCCTACACGACGCTCTTCCGATCTCTTCGCGCTTAATATGGACTAAAGGAGGCTTTT |
| F7 | ACACTCTTTCCCTACACGACGCTCTTCCGATCTACGTAGCTTTAATATGGACTAAAGGAGGCTTTT |
| F8 | ACACTCTTTCCCTACACGACGCTCTTCCGATCTATCCTATTTTAATATGGACTAAAGGAGGCTTTT |
| F9 | ACACTCTTTCCCTACACGACGCTCTTCCGATCTCAGGAGGCTTAATATGGACTAAAGGAGGCTTTT |
| F10 | ACACTCTTTCCCTACACGACGCTCTTCCGATCTCGACTGGGTTAATATGGACTAAAGGAGGCTTTT |
| F11 | ACACTCTTTCCCTACACGACGCTCTTCCGATCTCTTAAGATTTAATATGGACTAAAGGAGGCTTTT |
| F12 | ACACTCTTTCCCTACACGACGCTCTTCCGATCTGCAAGTAGTTAATATGGACTAAAGGAGGCTTTT |
|  |  |
| F13 | ACACTCTTTCCCTACACGACGCTCTTCCGATCTATATAGGATTAATATGGACTAAAGGAGGCTTTT |
| F14 | ACACTCTTTCCCTACACGACGCTCTTCCGATCTCACGTGTTTTAATATGGACTAAAGGAGGCTTTT |
| F15 | ACACTCTTTCCCTACACGACGCTCTTCCGATCTCGAACTGTTTAATATGGACTAAAGGAGGCTTTT |
| F16 | ACACTCTTTCCCTACACGACGCTCTTCCGATCTCTCTGTCTTTAATATGGACTAAAGGAGGCTTTT |
| F17 | ACACTCTTTCCCTACACGACGCTCTTCCGATCTGATGGAATTTAATATGGACTAAAGGAGGCTTTT |
| F18 | ACACTCTTTCCCTACACGACGCTCTTCCGATCTGGCAGACGTTAATATGGACTAAAGGAGGCTTTT |
| F19 | ACACTCTTTCCCTACACGACGCTCTTCCGATCTCACAGTTGTTAATATGGACTAAAGGAGGCTTTT |
| F20 | ACACTCTTTCCCTACACGACGCTCTTCCGATCTCCTTTACATTAATATGGACTAAAGGAGGCTTTT |
| F21 | ACACTCTTTCCCTACACGACGCTCTTCCGATCTCTAGTCATTTAATATGGACTAAAGGAGGCTTTT |
| F22 | ACACTCTTTCCCTACACGACGCTCTTCCGATCTGATCCAGCTTAATATGGACTAAAGGAGGCTTTT |
| F23 | ACACTCTTTCCCTACACGACGCTCTTCCGATCTGGATATGGTTAATATGGACTAAAGGAGGCTTTT |
| F24 | ACACTCTTTCCCTACACGACGCTCTTCCGATCTGTGACTACTTAATATGGACTAAAGGAGGCTTTT |
|  |  |
| F25 | ACACTCTTTCCCTACACGACGCTCTTCCGATCTCCTACAACTTAATATGGACTAAAGGAGGCTTTT |
| F26 | ACACTCTTTCCCTACACGACGCTCTTCCGATCTCTAGATTCTTAATATGGACTAAAGGAGGCTTTT |
| F27 | ACACTCTTTCCCTACACGACGCTCTTCCGATCTGAGTTAACTTAATATGGACTAAAGGAGGCTTTT |
| F28 | ACACTCTTTCCCTACACGACGCTCTTCCGATCTGGACGAGATTAATATGGACTAAAGGAGGCTTTT |
| F29 | ACACTCTTTCCCTACACGACGCTCTTCCGATCTGTCTACATTTAATATGGACTAAAGGAGGCTTTT |
| F30 | ACACTCTTTCCCTACACGACGCTCTTCCGATCTTATACCGTTTAATATGGACTAAAGGAGGCTTTT |
| F31 | ACACTCTTTCCCTACACGACGCTCTTCCGATCTCGTCGGCTTTAATATGGACTAAAGGAGGCTTTT |
| F32 | ACACTCTTTCCCTACACGACGCTCTTCCGATCTGAGAACTCTTAATATGGACTAAAGGAGGCTTTT |
| F33 | ACACTCTTTCCCTACACGACGCTCTTCCGATCTGCTGGCGATTAATATGGACTAAAGGAGGCTTTT |
| F34 | ACACTCTTTCCCTACACGACGCTCTTCCGATCTGTCCATTATTAATATGGACTAAAGGAGGCTTTT |
| F35 | ACACTCTTTCCCTACACGACGCTCTTCCGATCTTAGTCACATTAATATGGACTAAAGGAGGCTTTT |
| F36 | ACACTCTTTCCCTACACGACGCTCTTCCGATCTTGACGCATTTAATATGGACTAAAGGAGGCTTTT |
|  |  |
| F37 | ACACTCTTTCCCTACACGACGCTCTTCCGATCTGACGTCAATTAATATGGACTAAAGGAGGCTTTT |
| F38 | ACACTCTTTCCCTACACGACGCTCTTCCGATCTGCTCAGTTTTAATATGGACTAAAGGAGGCTTTT |
| F39 | ACACTCTTTCCCTACACGACGCTCTTCCGATCTGTAGAGCTTTAATATGGACTAAAGGAGGCTTTT |
| F40 | ACACTCTTTCCCTACACGACGCTCTTCCGATCTTAGCTAGTTTAATATGGACTAAAGGAGGCTTTT |
| F41 | ACACTCTTTCCCTACACGACGCTCTTCCGATCTTGAATTCGTTAATATGGACTAAAGGAGGCTTTT |
| F42 | ACACTCTTTCCCTACACGACGCTCTTCCGATCTTTCCTCACTTAATATGGACTAAAGGAGGCTTTT |
| F43 | ACACTCTTTCCCTACACGACGCTCTTCCGATCTGCGTTTCGTTAATATGGACTAAAGGAGGCTTTT |
| F44 | ACACTCTTTCCCTACACGACGCTCTTCCGATCTGTACTTGCTTAATATGGACTAAAGGAGGCTTTT |
| F45 | ACACTCTTTCCCTACACGACGCTCTTCCGATCTTACTGCGCTTAATATGGACTAAAGGAGGCTTTT |
| F46 | ACACTCTTTCCCTACACGACGCTCTTCCGATCTTCGGTACCTTAATATGGACTAAAGGAGGCTTTT |
| F47 | ACACTCTTTCCCTACACGACGCTCTTCCGATCTTTAAACAGTTAATATGGACTAAAGGAGGCTTTT |
| F48 | ACACTCTTTCCCTACACGACGCTCTTCCGATCTAATGCTGATTAATATGGACTAAAGGAGGCTTTT |
|  |  |
| F49 | ACACTCTTTCCCTACACGACGCTCTTCCGATCTGGTCTGACTTAATATGGACTAAAGGAGGCTTTT |
| F50 | ACACTCTTTCCCTACACGACGCTCTTCCGATCTTACGAATCTTAATATGGACTAAAGGAGGCTTTT |
| F51 | ACACTCTTTCCCTACACGACGCTCTTCCGATCTTCGCGTACTTAATATGGACTAAAGGAGGCTTTT |
| F52 | ACACTCTTTCCCTACACGACGCTCTTCCGATCTTGTGCTATTTAATATGGACTAAAGGAGGCTTTT |
| F53 | ACACTCTTTCCCTACACGACGCTCTTCCGATCTAATCACACTTAATATGGACTAAAGGAGGCTTTT |
| F54 | ACACTCTTTCCCTACACGACGCTCTTCCGATCTAGGTCAGTTTAATATGGACTAAAGGAGGCTTTT |
| F55 | ACACTCTTTCCCTACACGACGCTCTTCCGATCTGTTTCACTTTAATATGGACTAAAGGAGGCTTTT |
| F56 | ACACTCTTTCCCTACACGACGCTCTTCCGATCTTCCTACTATTAATATGGACTAAAGGAGGCTTTT |
| F57 | ACACTCTTTCCCTACACGACGCTCTTCCGATCTTGTAGGTCTTAATATGGACTAAAGGAGGCTTTT |
| F58 | ACACTCTTTCCCTACACGACGCTCTTCCGATCTAAGATTGCTTAATATGGACTAAAGGAGGCTTTT |
| F59 | ACACTCTTTCCCTACACGACGCTCTTCCGATCTAGGCAATGTTAATATGGACTAAAGGAGGCTTTT |
| F60 | ACACTCTTTCCCTACACGACGCTCTTCCGATCTATTGCATCTTAATATGGACTAAAGGAGGCTTTT |
|  |  |
| F61 | ACACTCTTTCCCTACACGACGCTCTTCCGATCTTCCAGCCTTTAATATGGACTAAAGGAGGCTTTT |
| F62 | ACACTCTTTCCCTACACGACGCTCTTCCGATCTTGGTCTTCTTAATATGGACTAAAGGAGGCTTTT |
| F63 | ACACTCTTTCCCTACACGACGCTCTTCCGATCTAAGCGGTCTTAATATGGACTAAAGGAGGCTTTT |
| F64 | ACACTCTTTCCCTACACGACGCTCTTCCGATCTAGAACACCTTAATATGGACTAAAGGAGGCTTTT |
| F65 | ACACTCTTTCCCTACACGACGCTCTTCCGATCTATGCATCCTTAATATGGACTAAAGGAGGCTTTT |
| F66 | ACACTCTTTCCCTACACGACGCTCTTCCGATCTCCATACACTTAATATGGACTAAAGGAGGCTTTT |
| F67 | ACACTCTTTCCCTACACGACGCTCTTCCGATCTTGCGGTTATTAATATGGACTAAAGGAGGCTTTT |
| F68 | ACACTCTTTCCCTACACGACGCTCTTCCGATCTAACCGTGTTTAATATGGACTAAAGGAGGCTTTT |
| F69 | ACACTCTTTCCCTACACGACGCTCTTCCGATCTACTCTAAGTTAATATGGACTAAAGGAGGCTTTT |
| F70 | ACACTCTTTCCCTACACGACGCTCTTCCGATCTATGAGGAATTAATATGGACTAAAGGAGGCTTTT |
| F71 | ACACTCTTTCCCTACACGACGCTCTTCCGATCTCCAGCACGTTAATATGGACTAAAGGAGGCTTTT |
| F72 | ACACTCTTTCCCTACACGACGCTCTTCCGATCTCGCTTCTGTTAATATGGACTAAAGGAGGCTTTT |
|  |  |
| R1 | CTCGGCATTCCTGCTGAACCGCTCTTCCGATCTTATCTCCGTCGAATTCAAGCTTAGATCTGATA |
| R2 | CTCGGCATTCCTGCTGAACCGCTCTTCCGATCTTGATCCGATCGAATTCAAGCTTAGATCTGATA |
| R3 | CTCGGCATTCCTGCTGAACCGCTCTTCCGATCTAGTAGTGGTCGAATTCAAGCTTAGATCTGATA |
| R4 | CTCGGCATTCCTGCTGAACCGCTCTTCCGATCTCAATCATCTCGAATTCAAGCTTAGATCTGATA |
| R5 | CTCGGCATTCCTGCTGAACCGCTCTTCCGATCTGAACGCTGTCGAATTCAAGCTTAGATCTGATA |
| R6 | CTCGGCATTCCTGCTGAACCGCTCTTCCGATCTGCGGCGAATCGAATTCAAGCTTAGATCTGATA |
| R7 | CTCGGCATTCCTGCTGAACCGCTCTTCCGATCTGTGGGATATCGAATTCAAGCTTAGATCTGATA |
| R8 | CTCGGCATTCCTGCTGAACCGCTCTTCCGATCTTCATTAGGTCGAATTCAAGCTTAGATCTGATA |
| R9 | CTCGGCATTCCTGCTGAACCGCTCTTCCGATCTACAGTGCATCGAATTCAAGCTTAGATCTGATA |
| R10 | CTCGGCATTCCTGCTGAACCGCTCTTCCGATCTAGTTGCTATCGAATTCAAGCTTAGATCTGATA |
| R11 | CTCGGCATTCCTGCTGAACCGCTCTTCCGATCTCGGACGTGTCGAATTCAAGCTTAGATCTGATA |
| R12 | CTCGGCATTCCTGCTGAACCGCTCTTCCGATCTGACACTCTTCGAATTCAAGCTTAGATCTGATA |
| R13 | CTCGGCATTCCTGCTGAACCGCTCTTCCGATCTGGCGAGGATCGAATTCAAGCTTAGATCTGATA |
| R14 | CTCGGCATTCCTGCTGAACCGCTCTTCCGATCTGTTGTCCCTCGAATTCAAGCTTAGATCTGATA |
| R15 | CTCGGCATTCCTGCTGAACCGCTCTTCCGATCTTTCTGATGTCGAATTCAAGCTTAGATCTGATA |
| R16 | CTCGGCATTCCTGCTGAACCGCTCTTCCGATCTACCGTTATTCGAATTCAAGCTTAGATCTGATA |
| R17 | CTCGGCATTCCTGCTGAACCGCTCTTCCGATCTCCGGATAGTCGAATTCAAGCTTAGATCTGATA |
| R18 | CTCGGCATTCCTGCTGAACCGCTCTTCCGATCTCGGTTGATTCGAATTCAAGCTTAGATCTGATA |
| R19 | CTCGGCATTCCTGCTGAACCGCTCTTCCGATCTGCAGCCTCTCGAATTCAAGCTTAGATCTGATA |
| R20 | CTCGGCATTCCTGCTGAACCGCTCTTCCGATCTGGTCCTTGTCGAATTCAAGCTTAGATCTGATA |
| R21 | CTCGGCATTCCTGCTGAACCGCTCTTCCGATCTTGAGAGTGTCGAATTCAAGCTTAGATCTGATA |
| R22 | CTCGGCATTCCTGCTGAACCGCTCTTCCGATCTTTGAGTGTTCGAATTCAAGCTTAGATCTGATA |
| R23 | CTCGGCATTCCTGCTGAACCGCTCTTCCGATCTCAAGACCATCGAATTCAAGCTTAGATCTGATA |
| R24 | CTCGGCATTCCTGCTGAACCGCTCTTCCGATCTCCGTCTGATCGAATTCAAGCTTAGATCTGATA |
| R25 | CTCGGCATTCCTGCTGAACCGCTCTTCCGATCTTGCGGTTATCGAATTCAAGCTTAGATCTGATA |
| R26 | CTCGGCATTCCTGCTGAACCGCTCTTCCGATCTAACCGTGTTCGAATTCAAGCTTAGATCTGATA |
| R27 | CTCGGCATTCCTGCTGAACCGCTCTTCCGATCTACTCTAAGTCGAATTCAAGCTTAGATCTGATA |
| R28 | CTCGGCATTCCTGCTGAACCGCTCTTCCGATCTATGAGGAATCGAATTCAAGCTTAGATCTGATA |
| R29 | CTCGGCATTCCTGCTGAACCGCTCTTCCGATCTCCAGCACGTCGAATTCAAGCTTAGATCTGATA |
| R30 | CTCGGCATTCCTGCTGAACCGCTCTTCCGATCTCGCTTCTGTCGAATTCAAGCTTAGATCTGATA |
| R31 | CTCGGCATTCCTGCTGAACCGCTCTTCCGATCTTCCAGCCTTCGAATTCAAGCTTAGATCTGATA |
| R32 | CTCGGCATTCCTGCTGAACCGCTCTTCCGATCTTGGTCTTCTCGAATTCAAGCTTAGATCTGATA |
| R33 | CTCGGCATTCCTGCTGAACCGCTCTTCCGATCTAAGCGGTCTCGAATTCAAGCTTAGATCTGATA |
| R34 | CTCGGCATTCCTGCTGAACCGCTCTTCCGATCTAGAACACCTCGAATTCAAGCTTAGATCTGATA |
| R35 | CTCGGCATTCCTGCTGAACCGCTCTTCCGATCTATGCATCCTCGAATTCAAGCTTAGATCTGATA |
| R36 | CTCGGCATTCCTGCTGAACCGCTCTTCCGATCTCCATACACTCGAATTCAAGCTTAGATCTGATA |
| R37 | CTCGGCATTCCTGCTGAACCGCTCTTCCGATCTGTTTCACTTCGAATTCAAGCTTAGATCTGATA |
| R38 | CTCGGCATTCCTGCTGAACCGCTCTTCCGATCTTCCTACTATCGAATTCAAGCTTAGATCTGATA |
| R39 | CTCGGCATTCCTGCTGAACCGCTCTTCCGATCTTGTAGGTCTCGAATTCAAGCTTAGATCTGATA |
| R40 | CTCGGCATTCCTGCTGAACCGCTCTTCCGATCTAAGATTGCTCGAATTCAAGCTTAGATCTGATA |
| R41 | CTCGGCATTCCTGCTGAACCGCTCTTCCGATCTAGGCAATGTCGAATTCAAGCTTAGATCTGATA |
| R42 | CTCGGCATTCCTGCTGAACCGCTCTTCCGATCTATTGCATCTCGAATTCAAGCTTAGATCTGATA |
| R43 | CTCGGCATTCCTGCTGAACCGCTCTTCCGATCTGACGTCAATCGAATTCAAGCTTAGATCTGATA |
| R44 | CTCGGCATTCCTGCTGAACCGCTCTTCCGATCTGCTCAGTTTCGAATTCAAGCTTAGATCTGATA |
| R45 | CTCGGCATTCCTGCTGAACCGCTCTTCCGATCTGTAGAGCTTCGAATTCAAGCTTAGATCTGATA |
| R46 | CTCGGCATTCCTGCTGAACCGCTCTTCCGATCTTAGCTAGTTCGAATTCAAGCTTAGATCTGATA |
| R47 | CTCGGCATTCCTGCTGAACCGCTCTTCCGATCTTGAATTCGTCGAATTCAAGCTTAGATCTGATA |
| R48 | CTCGGCATTCCTGCTGAACCGCTCTTCCGATCTTTCCTCACTCGAATTCAAGCTTAGATCTGATA |
| R49 | CTCGGCATTCCTGCTGAACCGCTCTTCCGATCTCGTCGGCTTCGAATTCAAGCTTAGATCTGATA |
| R50 | CTCGGCATTCCTGCTGAACCGCTCTTCCGATCTGAGAACTCTCGAATTCAAGCTTAGATCTGATA |
| R51 | CTCGGCATTCCTGCTGAACCGCTCTTCCGATCTGCTGGCGATCGAATTCAAGCTTAGATCTGATA |
| R52 | CTCGGCATTCCTGCTGAACCGCTCTTCCGATCTGTCCATTATCGAATTCAAGCTTAGATCTGATA |
| R53 | CTCGGCATTCCTGCTGAACCGCTCTTCCGATCTTAGTCACATCGAATTCAAGCTTAGATCTGATA |
| R54 | CTCGGCATTCCTGCTGAACCGCTCTTCCGATCTTGACGCATTCGAATTCAAGCTTAGATCTGATA |
| R55 | CTCGGCATTCCTGCTGAACCGCTCTTCCGATCTGGTCTGACTCGAATTCAAGCTTAGATCTGATA |
| R56 | CTCGGCATTCCTGCTGAACCGCTCTTCCGATCTTACGAATCTCGAATTCAAGCTTAGATCTGATA |
| R57 | CTCGGCATTCCTGCTGAACCGCTCTTCCGATCTTCGCGTACTCGAATTCAAGCTTAGATCTGATA |
| R58 | CTCGGCATTCCTGCTGAACCGCTCTTCCGATCTTGTGCTATTCGAATTCAAGCTTAGATCTGATA |
| R59 | CTCGGCATTCCTGCTGAACCGCTCTTCCGATCTAATCACACTCGAATTCAAGCTTAGATCTGATA |
| R60 | CTCGGCATTCCTGCTGAACCGCTCTTCCGATCTAGGTCAGTTCGAATTCAAGCTTAGATCTGATA |
| R61 | CTCGGCATTCCTGCTGAACCGCTCTTCCGATCTGCGTTTCGTCGAATTCAAGCTTAGATCTGATA |
| R62 | CTCGGCATTCCTGCTGAACCGCTCTTCCGATCTGTACTTGCTCGAATTCAAGCTTAGATCTGATA |
| R63 | CTCGGCATTCCTGCTGAACCGCTCTTCCGATCTTACTGCGCTCGAATTCAAGCTTAGATCTGATA |
| R64 | CTCGGCATTCCTGCTGAACCGCTCTTCCGATCTTCGGTACCTCGAATTCAAGCTTAGATCTGATA |
