# Supplementary material for: Paths to adaptation under fluctuating nitrogen starvation: The spectrum of adaptive mutations in Saccharomyces cerevisiae is shaped by retrotransposons and microhomology-mediated recombination
Source: PLoS Genet. 2023 May 16;19(5):e1010747. doi: 10.1371/journal.pgen.1010747 (PMC10218751; doi:10.1371/journal.pgen.1010747)
Supplement: S2 Table — (DOCX) [file pgen.1010747.s008.docx]

| FOS1 | AATGATACGGCGACCACCGAGATCTACACTCTTTCCCTACACGACGCTCTTCCGATCT TTAATATGGACTAAAGGAGGCTTTT |
| --- | --- |
| FOS2 | AATGATACGGCGACCACCGAGATCTACACTCTTTCCCTACACGACGCTCTTCCGATCT TCGTTTAATATGGACTAAAGGAGGCTTTT |
| FOS3 | AATGATACGGCGACCACCGAGATCTACACTCTTTCCCTACACGACGCTCTTCCGATCT CTTATTAATATGGACTAAAGGAGGCTTTT |
| FOS4 | AATGATACGGCGACCACCGAGATCTACACTCTTTCCCTACACGACGCTCTTCCGATCT GCCGTTTAATATGGACTAAAGGAGGCTTTT |
| FOS5 | AATGATACGGCGACCACCGAGATCTACACTCTTTCCCTACACGACGCTCTTCCGATCT ATGATTTAATATGGACTAAAGGAGGCTTTT |
| FOS6 | AATGATACGGCGACCACCGAGATCTACACTCTTTCCCTACACGACGCTCTTCCGATCT ACTGCTTTAATATGGACTAAAGGAGGCTTTT |
| FOS7 | AATGATACGGCGACCACCGAGATCTACACTCTTTCCCTACACGACGCTCTTCCGATCT CGTTGATTAATATGGACTAAAGGAGGCTTTT |
| FOS8 | AATGATACGGCGACCACCGAGATCTACACTCTTTCCCTACACGACGCTCTTCCGATCT CAGCAGTTAATATGGACTAAAGGAGGCTTTT |
| FOS9 | AATGATACGGCGACCACCGAGATCTACACTCTTTCCCTACACGACGCTCTTCCGATCT TTCAGCTTAATATGGACTAAAGGAGGCTTTT |
| FOS10 | AATGATACGGCGACCACCGAGATCTACACTCTTTCCCTACACGACGCTCTTCCGATCT GTAACCTTAATATGGACTAAAGGAGGCTTTT |
| ROS1 | CAAGCAGAAGACGGCATACGAGATCGGTCTCGGCATTCCTGCTGAACCGCTCTTCCGATCT TCGAATTCAAGCTTAGATCTGATA |
| ROS2 | CAAGCAGAAGACGGCATACGAGATCGGTCTCGGCATTCCTGCTGAACCGCTCTTCCGATCT AGTCGAATTCAAGCTTAGATCTGATA |
| ROS3 | CAAGCAGAAGACGGCATACGAGATCGGTCTCGGCATTCCTGCTGAACCGCTCTTCCGATCT CTTATCGAATTCAAGCTTAGATCTGATA |
| ROS4 | CAAGCAGAAGACGGCATACGAGATCGGTCTCGGCATTCCTGCTGAACCGCTCTTCCGATCT GACTCGAATTCAAGCTTAGATCTGATA |
| ROS5 | CAAGCAGAAGACGGCATACGAGATCGGTCTCGGCATTCCTGCTGAACCGCTCTTCCGATCT ACATCGAATTCAAGCTTAGATCTGATA |
| ROS6 | CAAGCAGAAGACGGCATACGAGATCGGTCTCGGCATTCCTGCTGAACCGCTCTTCCGATCT CACGTCGAATTCAAGCTTAGATCTGATA |
