# Supplementary material for: Paths to adaptation under fluctuating nitrogen starvation: The spectrum of adaptive mutations in Saccharomyces cerevisiae is shaped by retrotransposons and microhomology-mediated recombination
Source: PLoS Genet. 2023 May 16;19(5):e1010747. doi: 10.1371/journal.pgen.1010747 (PMC10218751; doi:10.1371/journal.pgen.1010747)
Supplement: Attachment — Submitted filename: 130653_1_rebuttal_2571865_rgp9hk (1).pdf [file pgen.1010747.s013.pdf]

Point by Point Description of Revisions

We thank the reviewers for their time, effort and constructive input. Below, our responses are bolded with yellow highlighting, while the reviewers' comments are italicized.

Reviewer #1 (Evidence, reproducibility and clarity (Required)):

Summary

The manuscript by Hays and colleagues described the spectrum of mutations that drive adaptation in nitrogen-limit environment by experimental evolution. The approach of serial transfer (fluctuating condition) allowed them to find that Ty insertion is the major mutation type for adaptive evolution. This was neither observed in nitrogen-limited condition when another experimental evolution approach, chemostat (non-fluctuating condition), was applied, nor in glucose-limited condition. The authors concluded that not only selection pressure itself but also how selection is applied are important to shape the adaptive events.

Major points

Both serial transfer and chemostat are commonly used approaches of experimental evolution. In the manuscript, the authors refer serial transfer to "fluctuating" condition because the low nitrogen source would be consumed to none during the interval of transfers. I am wondering whether the authors have estimated the nitrogen uptake (consumption) during the transfer intervals and whether the nitrogen was exhausted within 48 hours.

We appreciate the reviewer's question, and although we did not directly measure nitrogen consumption throughout this specific experiment, ammonium was the limiting nutrient in the defined medium which has been previously used to achieve transient nitrogen starvation conditions in other yeast experimental evolutions (Blundell et al. 2019). In that previous work, it was confirmed that addition of ammonium above 0.04% (up to 0.15%) led to additional rounds of doubling – confirming that the amount of ammonium provided was in fact the limiting nutrient. Finally, we point out that the adaptive mutations recovered in this study predominantly impact genes known to affect nitrogen catabolism, as is expected under nitrogen-limited evolution conditions.

We've updated the methods section to ensure the rationale for this medium choice is clearly stated.

Since this is not precisely controlled by experiment design, the "fluctuating" condition itself may be not stable during the long-term evolution. For example, as population evolved, the rate and the amount of nitrogen uptake might change. I feel a better experiment setup for "fluctuating" condition is like 24 hour "low-nitrogen (ammonium)" - 24 hour "no ammonium" and so on. If the adaptive mutations (e.g. adaptive Ty) specifically respond to such "fluctuating" condition rather than chemostat, the authors can measure their fitness in nitrogen starvation condition, which is expected to be fitter than mutants observed only in chemostat (e.g. copy number variation of nitrogen transporters).

The reviewer correctly points out that nitrogen availability will change as the population adapts, and it is likely that some portion of the population become better at utilizing the newly available nitrogen upon transfer into fresh medium over time. This is in fact the intention of this experimental design. We have rephrased the text of the main paper to emphasize that our fluctuating conditions represent fluctuations in the nutrient availability in fresh medium upon transfer, and not strict oscillating nitrogen concentrations that cells experience locally throughout all generations.

We note that in the reviewer-proposed experimental design (using 2 stages of low- and no-nitrogen media), that the low-nitrogen condition would still exhibit the same population-dependent nitrogen usage dynamics as the population adapts over time. We chose our evolution conditions to apply a selective pressure for cells to become best adapted to the environmental fluctuations associated with this transfer regimen, and we have updated the main paper to clarify this point. We thank the reviewer for helping us clarify this important point.

The authors compared their results with published dataset using nitrogen-limitation chemostat and the mutation spectrum is different. In addition to the "fluctuating" and "non-fluctuating" difference as mentioned above, other factors need to be considered. First, the nitrogen-limited conditions in the two studies are different. The authors used 0.04% ammonium sulfate while Hong et al used "800 uM nitrogen regardless of the molecular form of the nitrogen", which may influence the mutation spectrum and need to be discussed. Second, bottlenecks were applied for each transfer in this study, in comparison with constant population size in chemostat, which will influence the efficiency of selection and further the evolutionary dynamics and outcomes. Thus, population size and bottlenecks need to take in to account to make comparisons of mutation spectrum.

We thank the reviewer for their point: we have expanded the section of the main text addressing the differences in how serial transfer and chemostat conditions are applied, the media differences necessitated by such and specifically how the conditions between our study and the Hong *et al* study differ. We believe the additional detail now better highlights our point that how selection is applied shapes adaptive events, and we thank the reviewer for their helpful input.

The authors found that Ty mutagenesis accounts for a substantial number of adaptive mutations in nitrogen limitation. I am wondering for adaptive clones, whether Ty occurred independently or is more likely to co-exist with other drivers.

We appreciate the reviewer's question. In the clones with adaptive Ty insertions, the only co-occurring adaptive mutation is autodiploidization. There were no additional mutational classes that were adaptive and co-occur with adaptive Ty insertions in our dataset. However, many novel Ty insertions are neutral, and these DO co-occur with beneficial mutations. These data are captured in Figure 5A, and in detail in Supplemental File 1. The blue bar in the adaptive haploids reflect neutral-fitness Ty insertions that co-occur with other mutations that drive fitness increase. These are distinct from the Ty insertions that are themselves responsible for the fitness increase, which are captured in the orange bar. We have clarified the text surrounding the Fig 5A results to better emphasize these findings.

What is the distribution of number of clones with one, two, and multiple mutations? If there is co-existence of driver mutations, what is the relative contribution of each to adaptation? The phenotypic validation of Ty mutagenesis for adaptation is expected while it seems only one case was presented in Figure 2 (mep1Ty−731427).

Aside from diploidization events, only one clone with two nitrogen-adaptive mutations was identified in this study: a double mutant with mutations in both *gat1* and *tor1*. Please see Supplemental File 1 (which is sortable) for a complete outline of all clones with mutations and fitness remeasurements. In the case of diploids that have additional beneficial mutations, those data are shown in Figure 3 with diploids indicated as well as the ploidy of the secondary beneficial mutation, and again in detail in Supplemental File 1.
The reviewer is correct in that only one Ty mutation was dissected and validated in Figure 2. However, we inferred adaptation by Ty insertion through the observation of parallel adaptation, and we fitness remeasurements of many independent Ty insertion mutants.

Statistical analysis needs to be reinforced in the manuscript, including but not limited to Figure 2 fitness comparison among clones with different genotypes, Figure 5 Ty enrichment comparison, etc.

We thank the reviewer for their helpful suggestion. We have updated figures and figure legends to more clearly include statistical comparisons between genotypes for Figures 2 and 5: specifically describing the analyses used and the associated p-values for differences between WT and adaptive alleles and significance of Ty class enrichments.

Minor points

We thank the reviewer for their detailed and careful edits below and have addressed them in the main text and figures as applicable.

"For diploids, we only sequenced those with estimated fitness greater than diploidy alone would provide."

Main text clarified with additional explanation

"either through impacting alternate start (green triangle) or alternate stop sites (yellow and red triangles)." I do not see yellow and red triangles in Fig. 3. **Legend updated to reflect current figure color palette.**

Fig.2. FCY2 mutant fitness can be added as well?

Unfortunately, data for *FCY2* backcrossed mutants were not generated

"while we found only 212 novel Ty insertions in 488 glucose evolved clones (Figure 5B)" The value in the text does not match the one in the figure.

We appreciate the reviewer's attention to detail and have corrected the main text to match the correct value in Fig 5B.

In addition to adaptive Ty insertion, what is the genome-wide distribution or characteristics of other Ty, especially for nitrogen-limited condition? Is that distinct from glucose-limited condition?

Figure S5 addresses the major locations of Ty insertions upstream of tRNA genes, in both Glucose and Nitrogen limited evolutions, the insertion location previously published to be preferred; the only difference between glucose and nitrogen is that there are more in the nitrogen limited condition, though the profile of insertions upstream of tRNAs is essentially the same. In addition to insertions upstream on tRNAs, all other specific insertion locations are available in Supplemental File 1 and Supplemental File 4.

"Studies determining at which step(s) of the Ty life cycle nitrogen starvation shapes ty activity would be needed to determine the specific mechanism underlying the increase in transposon insertions." Here "ty" => "Ty"

Corrected! We thank the reviewer for their detailed reading.

Reviewer #1 (Significance (Required)):

The manuscript is a follow-up work of Levy et al. 2015 and Blundell et al. 2019. In general, the research is interesting and point out the important role of Ty for adaptive evolution in nitrogen-limited environment. It also compared the spectrum of adaptive mutations in response to nitrogen limitation by serial transfer (this work) and chemostat (especially the work of Gresham lab). The paper is well-written as well. Audience from the field of genetics, genomics and evolution will be interested in this work.
My field of expertise: genetics, experimental evolution, budding yeast

We thank the reviewer for their kind comments, constructive input, and generosity with their time.

Reviewer #2 (Evidence, reproducibility and clarity (Required)):

Hays et al. sequence and analyze the mutational spectrum from a set of S. cerevisiae strains evolved in a nitrogen limiting environment, and detail genes that recurrently are found to be mutated in a fluctuating nitrogen limiting environment. These data are contrasted to evolution under glucose limited environments and non-fluctuating environments. Specifically, Hays et al. observe a high proportion of Ty element-mediated mutations arising from strains evolved under the fluctuating nitrogen limiting regime. Their fitness data are robust and clearly demonstrate that these mutations reproducibly lead to improved fitness under nitrogen limitation (based on the authors' defined criteria). Overall, the observed bias of the high proportion of Ty-mediated mutation in fluctuating nitrogen starvation is unexpected and an important finding. Further, the discussion was thoughtful and well executed in detailing interpretations of the data more broadly. We are generally positive about this work and find the analyses robust and convincing. The authors should address the concerns listed below prior to acceptance/publication.

We thank the reviewer for their kind words and enthusiasm for our study, we have worked to address their constructive feedback as detailed below.

Reviewer #2 (Significance (Required)):

Major comments to be addressed:

The claim that the 3' UTR Ty insertions in MEP1 are apparently gain of function is very interesting. The authors should consider performing RT-PCR or strand specific RNAseq to see whether the antisense transcript is reduced and the MEP1 transcript is increased in the presence of the 3' UTR insertion. This would provide much stronger support for their claim that MEP1 3' Ty insertions are gain of function. Orientation information is critical to provide!

We agree that these future directions are exciting and of extreme interest! We however believe they are out of the scope of this current study which already includes substantial data and analysis. We note that we did not claim that the 3' UTR insertions are gain of function – instead, we suggested that "Ty insertions in the 3' region unique to the MEP1 locus may affect fitness in nitrogen limitation via a mechanism different than the putative gain of function missense mutations in the coding region itself". We did not speculate on the mechanism by which these insertions are adaptive, but it is an active line of research and we look forward to discovering the mechanism.

The authors seemed to miss a golden opportunity to measure Ty1 expression or transposition under fluctuating/non-fluctuating nitrogen starvation. Otherwise, the claims of increased Ty activity are unsupported. The authors measured an endpoint (Ty insertion), but this says nothing directly as to the rate of activity, although it is presumably correlated. However, based on the data one could argue activity may be equal in all environments, but the mutational events caused by Ty activity are uniquely selected for in fluctuating nitrogen starvation. As it stands, either model (increased activity vs. differential strength of selection) are equally likely. At a minimum, the authors should at least address this point.

We appreciate the reviewer bringing this concern to our attention: we address the reviewer's concerns in 3 ways: First, we've rephrased to more explicitly consider the possibility that the observed difference in novel Ty insertions could be driven at the level of selection, not activity. Second, we've clarified the main text to greater emphasize our reasoning for why we speculate the inference of greater Ty activity under nitrogen starvation may be more likely based on the level of

presumptive neutral Ty insertions being greater in nitrogen than in glucose (even after normalization for the number of evolved generations). Third, we've performed additional experiments that support that, at least with an artificial retrotransposition reporter construct, these starvation conditions show additional Ty activity in nitrogen compared to glucose (note, we have not carried out such experiments in chemostats, and do not currently have a functioning chemostat set up). We're including these results below, though have not included them in the manuscript, as we intend to generate additional data for a subsequent study to make these claims more robust. We feel that adding them to this manuscript would make it less focused.

To assess Ty activity in yeast experiencing different nutrient conditions, we used a modified version of a plasmid-based Ty reporter created previously by Curcio and Garfinkel, 1991, PNAS 88(3):936-40. The original reporter construct used an inducible GAL promoter to initiate Ty transcription from the plasmid, and new Ty insertions confer the ability for the strain to grow on SC-His. To assess Ty activity induced by nitrogen limitation, we excised the GAL promoter and instead used the native Ty promoter from the insertion found at YPLWTy1-1. This Ty promoter was selected based on having recovered novel Ty insertions in evolved clones that originated from this locus. Plasmid pGS234 was created by replacing the promoter containing XhoI fragment from pGTy1mhis3-AI with XhoI fragment containing promoter from chromosomal location of YPLWTy1-1.
Strains bearing the Ty reporter plasmid pGS234 were subjected to nitrogen limited media and glucose limited media to assess transposon activity in these conditions. We observe significantly more Ty activity from the reporter plasmid in nitrogen-limited conditions than in glucose limited conditions or in SC-ura medium (see Figure below).

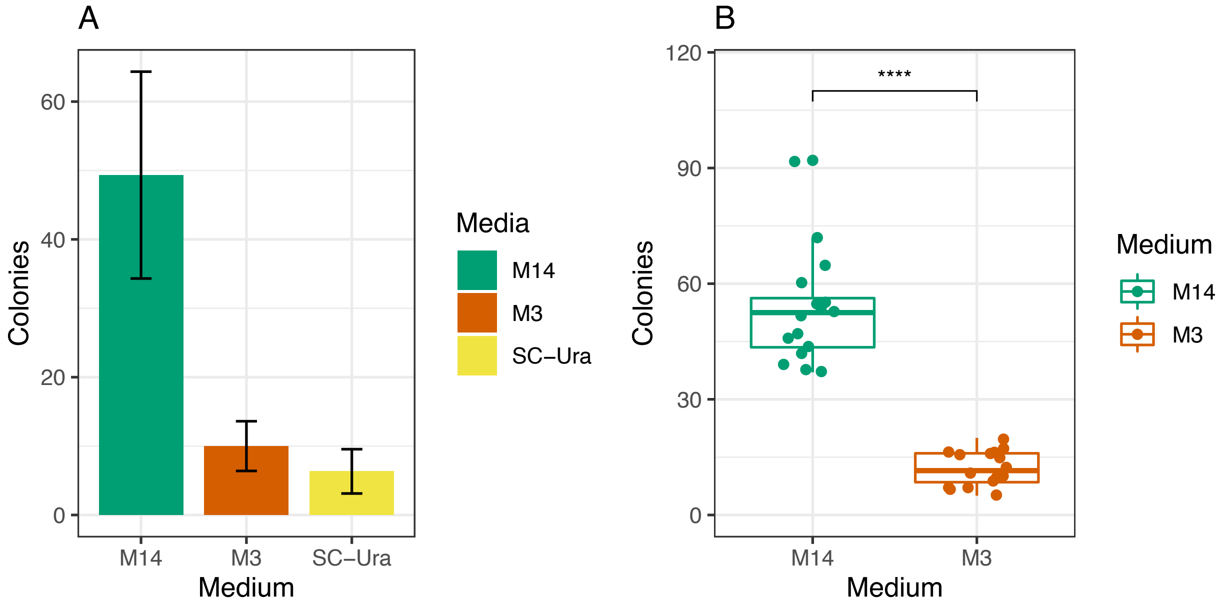

Panel A: Bars represent average of three WT strains with transposon reporter plasmid; each value is number of colonies on SC-His medium with each His+ colony representing independent Ty transposition events. Strains were grown in SC-Ura and then shifted to M14, M3 or SC-Ura as a control for 48 hours and plated on SC-His plates.

Panel B. One WT strain with pGS234 was subjected to a fluctuation test (16x 5ml tubes) in M14 and M3 media. Each dot represents the number of colonies on each SC-His plate. Kruskal-Wallis chi-squared = 23.341, df = 1, p-value = 1.357e-06

In line with the above, we think the authors should soften some points in the discussion as it stands. For example: "The significant increase of Ty activity under this specific fluctuating nitrogen-starvation..." We feel the data does not exclusively support increased activity of Ty, that would require the aforementioned assays. As it stands, we feel this is more appropriate: ": "The significant increase of Ty insertions under this specific fluctuating nitrogen-starvation..."

We edited the main text to include this suggested language change.

Minor comments to be addressed:

Please provide a citation for the following statement "The single copy of Ty5 in the ancestor is known to be inactive and gives rise to no new insertions under either glucose or nitrogen limitation" - Voytas & Boeke. Nature 1992.

We appreciate the reviewer catching this, and the reference has been added.

We found the following to be a confusing sentence: "Indeed, if global Ty derepression reflects a host-parasite coevolution that minimizes host cost and maximizes potential for survival of both, the role of transposons in host evolvability is important (Levin and Moran 2011)."

We have clarified this sentence by editing it to: "Indeed, the role of transposons in host evolvability is important: global Ty derepression could reflect host-parasite coevolution towards a less parasitic lifestyle: resulting in minimal host cost and maximized potential for survival of both, especially under detrimental environmental conditions (Levin and Moran 2011)"

Reviewer #3 (Evidence, reproducibility and clarity (Required)):

Hays et al. studied the genomic changes that lead to adaptation under fluctuating nitrogen starvation. In addition to loss of function alleles, the authors identified adaptive gain-of- function alleles. Furthermore, their results demonstrate that Ty and microhomology-facilitated mutations in several candidate genes contribute substantially (though not exclusively) to the adaptation under nitrogen-limited serial transfer. Importantly, a novel lineage tracking method provides high resolution fitness measurements.

We appreciate the reviewer's helpful edits in clarifying and improving the manuscript, and appreciate their time and constructive input.

Despite the clear merits of the study, we also have a few relatively minor questions and suggestions

1. Please elaborate on the criteria they used to identify adaptive loci. The fact that these mutations occurred repeatedly is highlighted on Table 1, but perhaps numbers could also be included in the text, to increase clarity.
We have added the pertinent numbers to the main text to accompany the values captured in Table 1 and Supplemental file 1 and further emphasize selection criteria outline in the main text.

2. "Were also validated to a fitness effect of >0.01 in nitrogen-limited media". More details about the selection of this cut-off value need to be provided in either the text or the Methods section to increase clarity.

We agree and have clarified the limit of detection used in the methods section.

3. In Figure 3 it seems that the type of observed mutations was less important compared to the gene where the mutation occurred. Therefore, it seems that some genes, e.g. GAT1, contribute more to the observed fitness change. It would be beneficial if the authors discussed this observation.
We thank the reviewer for their observation and have included some additional discussion in the main text around the per-locus fitness observations as shown in Figure 3.

4. What was the reason to select samples from the 88th generation for glucose and from the 192nd generation for nitrogen, as presented in Figure 5? How does this affect the observations?

We thank the reviewer for their question: these generations were determined to best capture peak adaptive diversity (as discussed in Blundell et al 2019), based on population barcode dynamics in the original evolutions (Levy et al 2015, Blundell et al. 2019). The challenge is balancing picking a time point late enough, such that there are sufficient numbers of adaptive clones within independent lineages, yet early enough that few mutations have occurred (ideally only a single adaptive mutation per sequenced clone) and that no very fit clones have taken over the population. Because the fitness effects of beneficial mutations in glucose limited media were larger than in nitrogen limited media it was necessary to choose a later timepoint in the Nitrogen limited evolutions, to allow for there to be a sufficient fraction of the population carrying adaptive mutations. We believe this peak diversity makes these samples the most relevant for broadly assessing the adaptive mutational spectra.

5. The use of statistics is not always clear. Please provide a clear indication of the statistical methods/tests used, eg for Figure 5.
We thank the reviewer for this important point and have updated figures 2 and 5 and their corresponding legends for clarity surrounding statistical analysis used.

6. The authors could include a supplementary Table, summarising their findings on GAT1 locus, since the text is extensive and it is difficult to put all the information into perspective.

We note that row one of Table 1 in the main text is exactly this overview of the mutations observed at the *GAT1* locus. These mutations plus specific location and their fitness remeasurements are shown in Figure 3 panel A, and detailed descriptions of the mutations for each clone are also available in the sortable table in Supplemental File 1. For these reasons we've not included an additional *GAT1*-specific table.

7. The introduction is extremely detailed and informative, but at the same time quite lengthy; shortening it and only keeping the most relevant parts may increase readability.
We appreciate the reviewer's perspective but have not made substantial changes to remove information from the introduction as we feel that each of the subsections of the introduction are necessary to provide the appropriate context to the study.

8. More detailed figure legends (which should also include a brief mentioning of the statistics & sample size) would benefit comprehensibility. For example the black lines in Figure S4 are not described anywhere in the text.
We agree and have added further description of statistics used in legends throughout. Description of the black lines in Figure S4 has been included.

9. "Many of the 332 clones ... were beneficial" ◊ rephrase.
We have updated this sentence to clarify our intent.

Reviewer #3 (Significance (Required)):

Apart from the elegant characterization of adaptive mutations, perhaps the most important part of the study is that it highlights the importance of a particular selection regime. Together, the findings extend our knowledge on this important topic.
