# Supplementary material for: Paths to adaptation under fluctuating nitrogen starvation: The spectrum of adaptive mutations in Saccharomyces cerevisiae is shaped by retrotransposons and microhomology-mediated recombination
Source: PLoS Genet. 2023 May 16;19(5):e1010747. doi: 10.1371/journal.pgen.1010747 (PMC10218751; doi:10.1371/journal.pgen.1010747)
Supplement: Attachment — Submitted filename: Response_to_Reviews.pdf [file pgen.1010747.s014.pdf]

We thank all three reviewers for a very positive review experience. The reviewers' suggestions greatly improved the paper and we are grateful for the constructive and encouraging input while crafting this manuscript.

Reviewer's Responses to Questions
Comments to the Authors:
Please note here if the review is uploaded as an attachment.
Reviewer #1: I already reviewed this paper (Reviewer 3) for Review Commons, so for my full review, I refer to that text.
I am happy with the author's response and changes to the manuscript. Apart from the changes in response to my own review, I think that the authors also responded very well to the (minor) comments of the other reviewers, in particular the relation between transposon activity and selection regime is a very interesting addition that may merit a bit more attention in the discussion and perhaps even the abstract since it goes to the heart of the paper's main goal (investigating if and how selection regime influences the molecular mechanisms of evolutionary adaptation).
kevin verstrepen

Thank you for the feedback – we agree these changes improved the manuscript and have updated the abstract to reflect this emphasis in the discussion section. Thank you for your time!

Reviewer #2: My concerns have been addressed.
Thank you for taking the time to review the manuscript again!

Reviewer #3: Rereview of Hays et al.

First things first. I love this paper and I think it's super interesting. The new data not included in the revision are interesting for sure. They would add some heat to this paper!
The authors appreciate the suggestion to include the additional materials provided in response to the prior review and those data are now included as supplemental information

The authors have improved the paper but have utterly failed on one aspect (I regret not having been more forceful about this on the first round!) and it really has to be fixed.

Major critique:

1. One of the types of researchers who are going to be eager to read this paper is the retrotransposonologists. And the authors' ignoring the recommendation to provide orientation information is simply unacceptable. When you describe a TE insertion it is one of the most fundamental aspects, as it governs the likelihood that adjacent gene sequences

will be newly transcribed. As shown by Valerie Williamson, Stuart Scherer and a legion of other Ty1-ologists, there in an enhancer near the "left end" of the Ty1 and it is a potent activator of flanking gene expression! That's just one of the reasons why this info is important in deducing the mechanisms involved in the adaptive and nonadaptive insertions.

The information regarding Ty insertion direction was already present in both Supplemental Files 2 and 4, as it is part of the output produced by RelocaTE2, in the form STRAND=+ or STRAND=-. The RelocaTE2 output is present as a single, semi-colon delimited string in one of the columns (which is the form that is output by the software) of those two Supplemental Files.

Thus, and I know this is painful because your team did not do it up front, but you really have to go back and update your figures and tables to provide the orientation information. At MINIMUM, the following Figures and tables need to be updated to provide orientation information for each Ty element described: Most especially Figure 3 (orientation relative to target gene transcription), Figure S5 (orientation relative to target gene transcription) and all supplemental Tables (orientation relative to chromosome coordinates)

We agree that this is a valuable suggestion. In the main text we've included a new section in the results detailing our Ty1/2 insertion orientation analysis, and that indicated that we don't see a signature of bias in the novel insertion's directionality. Because we see no bias in insertion direction, we did not add this information to Table 1, as it substantially complicates the table; however, we did indicate insertion direction in Figures 3, and S5, which makes it clear that there is no bias in the insertion direction.

2. Similarly in some of the tables saying just "Ty" is not acceptable, each Ty can be assigend to one of the families very easily since they have very different sequences. An exception to this is Ty1 and Ty2 which are similar (they are basically subfamilies), thus in these cases it is acceptable to list them as Ty1/Ty2

Thank you for helping us clarify our language; we have implemented the reviewer's suggestion throughout the main text, table 1 (which says Ty1/2) and in more detail in supplemental files 2 and 4. These changes disambiguate which families are being referenced, and address the specific instances mentioned by the reviewer below in minor critiques.

Minor critiques:
Below I list a whole slew of perhaps picayune critiques mostly of the transposon parts, please note they are aimed at making the intro and discussion part of your paper more accurate and hopefully also even more interesting.
The authors greatly appreciate the reviewer's attention to detail and agree that these language changes improve clarity and accuracy throughout. We're grateful for the time the

reviewer took to help improve this manuscript, and have implemented the changes suggested below.

2. Extremely picayune – but extremely annoying to reviewers and editors – you didn't put page numbers or line numbers. You will have to hunt
We've since added line and page numbers, and apologize for the previous oversight and inconvenience.
3. You may want to change the word "transposons" to "retrotransposons" in the title to avoid confusion. Many use the word transposon to refer to DNA transposons only. Yeast has none of the latter. You also use the terms transposition when retrotransposition would be more accurate throughout the text.
We've corrected to retrotransposition throughout.
4. Whenever possible do not use the term Ty which is collective. In almost all cases, use of the more specific term Ty1 (or Ty1/Ty2) is more specific and accurate
"including in Saccharomyces cerevisiae (many refs)". Please add temperature regulation: Pacquin and Willliamson PMID: 17815421, Boeke et al. PMID: 3025601, Lawler et al PMID: 11932388
We've specified Ty1/2 throughout unless referring collectively or generically to all Ty elements. The temperature regulation references have been added as well.
5. Table 1. Definitely specify Ty type – I expect they are all Ty1/2; ideally also specify orientation here. I am betting money that the MEP insertions will be interesting (i.e. non-random as to orientation). Same for figure 2 "Ty" allele, Fig. S4
We've specified Ty1/2 in the Table 1 header, but details about specific insertions are captured in detail in supplemental files 2 and 4. This includes both orientation as well as family. As comparatively few insertions are Ty2 and the insertion orientation does not seem to show bias, we've elected not to capture that in the summary Table1, but have added language to each locus section specifying novel Ty2 abundance. Ty orientation is addressed in a new results section, as detailed above following the reviewer's major critique. We have modified Figure 2 and made it clear in the legend to Figure S4 that it is both Ty1 and Ty2 insertions that are plotted.

6. *MEP1* section: The discussion about possible insertional interference with the MEP1 antisense RNA seems very plausible. Please state whether or not there is any evidence for similar 3' antisense RNAs for MEP2/3.

We now state that there is no evidence for such stable unannotated antisense transcripts for either *MEP2* or *MEP3*.

7. Incorrect sentence: "Others have observed Ty1 and Ty2 to be the most active classes of spontaneous Ty transposition (Curcio et al. 1990)". This sentence really isn't right. Curcio paper showed Ty1/2 were the only classes that could turn on gene expression. It turns out Ty3, 4 and 5 don't have this ability as they lack the Ty1/2 enhancer. I would simply say "Ty1

and Ty2 are far and away the most abundant classes of Ty element (PMID 9582191) and are highly active in yeast.

We've included this edit, and all of the following language edits as suggested as well, unless otherwise noted.

8. "or even disruption of the Ty elements themselves, some of which restrict other subclasses of Ty (Czaja et al. 2020)". This should be changed to Ty1 (twice).
9. "it will be interesting to understand the mechanism underlying this insertion-specific activation." To avoid confusion, when you are talking about pre-existing elements in the genome, as in this case, use the term "donor element-specific activation".
10. "as is the case with Ty activity under fluctuating nitrogen starvation". This is a golden opportunity to reference temperature control of Ty1 hopping.
11. Suggest change to "If cells experience increased mutation rates while under [specific] stress conditions"
12. Para that begins "Alternatively, rather than ...". Really worth mentioning here that in the wild, yeast likely overwinters in the guts of wasps. PMID: 22847440

We elected to not mention the overwintering of yeast in wasps. While we agree that it is an interesting and important part of yeast ecology, and likely exerts some interesting selection pressures, our data don't allow us to make any particular claims in this regard, and we felt that adding in something about overwintering would lessen the focus of the paper.

13. Methods: There is no such medium as SD–Ura, it is SC–Ura. Also, please be sure to use the minus sign and not the hyphen in this context!
14. Supp table 1. I believe that in column F, this is the authors' valiant attempt to identify the donor element that gave rise to the insertion. I think this is a dangerous game for two reasons. 1) Many Ty1 copies are identical in sequence and thus cannot be tracked. 2) Are you really sure that all the Ty1 sequences in the progenitor strain you worked with are identical in sequence and position to the SGD reference sequence, which I expect, is what was used to assign the Ty1 sequences? Probably better to be agnostic on this. Table needs a legend

We believe the reviewer is referring to supplemental files 2 and 4 rather than supplemental table 1 – the column F data of presumed subfamily are output by RelocaTE2, but we agree with the reviewer that these are unreliable given the nature of the short read data and the relatedness of subfamilies. As such we've deleted those columns from the RelocaTE2 output files. Legends for all the Supplemental Files are provided at the end of the manuscript document.
